# The Systemic Inflammation Response Index (SIRI) for Stratifying Mortality Risk in Multidrug‐Resistant Lower Respiratory Tract Infections Acquired in Hospital Settings

**DOI:** 10.1155/cjid/8850880

**Published:** 2026-09-10

**Authors:** Xia Hui, Xiao Ge, Yahui Liu, Jiebai Zhou, Yun Feng, Haixing Zhu

**Affiliations:** ^1^ Department of Respiratory and Critical Care Medicine, Huan County People’s Hospital, Qingyang, Gansu, China; ^2^ Department of Respiratory and Critical Care Medicine, Ruijin Hospital Affiliated Shanghai Jiao Tong University School of Medicine, Shanghai, China; ^3^ Institute of Respiratory Diseases, School of Medicine, Shanghai Jiao Tong University, Shanghai, China, sjtu.edu.cn; ^4^ Department of Respiratory and Critical Care Medicine, Zhongshan Hospital, Fudan University, Shanghai, China, fudan.edu.cn

**Keywords:** hospital-acquired infections, mortality, multidrug-resistant, prognosis, risk stratification, systemic inflammation response index (SIRI)

## Abstract

**Background:**

Timely risk assessment is important in the management of patients with multidrug‐resistant (MDR) hospital‐acquired lower respiratory tract infections (LRTI). The Systemic Inflammation Response Index (SIRI), an inflammatory biomarker derived from peripheral neutrophil, monocyte, and lymphocyte counts, has shown prognostic potential across several clinical settings. However, its prognostic value and clinically interpretable threshold in patients with MDR hospital‐acquired LRTI remain uncertain. This study aimed to evaluate the association between SIRI and in‐hospital mortality in this population.

**Methods:**

This single‐center retrospective cohort study included 214 patients diagnosed with MDR hospital‐acquired LRTI, with all‐cause in‐hospital mortality as the primary outcome. Cox proportional hazards models were used to assess the prognostic value of SIRI. Restricted cubic splines were applied to examine potential nonlinear associations. A data‐driven cutoff was evaluated using Kaplan–Meier survival analysis and stratified receiver operating characteristic (ROC) analysis.

**Results:**

When analyzed as a dichotomized variable using the data‐driven cutoff, high SIRI was associated with increased in‐hospital mortality (hazard ratio [HR] = 2.93, 95% confidence interval [CI]: 1.66–5.14, *p* < 0.001). Restricted cubic spline analysis suggested a nonlinear relationship, with risk increasing around a SIRI value of 3 and then approaching a high‐risk plateau. Kaplan–Meier analysis showed lower survival among patients with SIRI > 3 (log‐rank *p* < 0.001). However, stratified ROC analyses showed only modest discrimination when SIRI was treated as a continuous variable within the low‐risk (SIRI ≤ 3, area under the curve [AUC] = 0.556) and high‐risk (SIRI > 3, AUC = 0.567) subgroups.

**Conclusion:**

SIRI may help identify a subgroup of patients with MDR hospital‐acquired LRTI who have a higher mortality risk, particularly when interpreted using a threshold of approximately 3.0. Given the modest ROC performance and retrospective single‐center design, this cutoff should be regarded as an exploratory risk‐stratification signal rather than a stand‐alone clinical decision tool. External prospective validation is required before clinical implementation.

## 1. Introduction

Hospital‐acquired lower respiratory tract infections (LRTI), particularly those caused by multidrug‐resistant (MDR) organisms, represent a formidable challenge in modern clinical practice, contributing significantly to patient morbidity, prolonged hospital stays, and mortality [1, 2]. The escalating prevalence of MDR pathogens, such as carbapenem‐resistant Enterobacteriaceae and *Acinetobacter baumannii*, complicates therapeutic management and is independently associated with increased mortality [3, 4]. This underscores the urgent need for accurate and early risk stratification to guide clinical decision‐making, optimize resource allocation, and improve patient outcomes [5, 6].

While established scoring systems like APACHE II and SOFA are valuable in the ICU setting, they can be complex to calculate and may not be universally applied outside of critical care [4]. Consequently, there is growing interest in simple, readily available biomarkers that reflect the host systemic inflammatory state. The Systemic Inflammation Response Index (SIRI) integrates neutrophil, monocyte, and lymphocyte counts and therefore captures both myeloid activation and adaptive immune suppression. Elevated SIRI has been associated with poor prognosis in malignancy and cardiovascular disease [7, 8], and recent studies have reported its association with infectious complications such as pneumonia after acute ischemic stroke, spontaneous intracerebral hemorrhage, and aneurysmal subarachnoid hemorrhage [9–11].

Despite these findings, the role of SIRI in patients with established MDR hospital‐acquired LRTI remains insufficiently defined. This population differs from general pneumonia or poststroke cohorts because MDR infection is often accompanied by delayed effective therapy, greater baseline illness severity, and complex immune dysregulation. Importantly, previous studies have not clarified whether SIRI should be interpreted as a continuous predictor or as a threshold‐based marker in this specific setting. The optimal cutoff and the shape of the dose–response relationship between SIRI and mortality also remain uncertain. Therefore, the primary objective of this study was to evaluate the prognostic utility of SIRI for all‐cause in‐hospital mortality among patients with MDR hospital‐acquired LRTI. As a secondary objective, we aimed to explore whether a clinically interpretable threshold could support preliminary risk stratification.

## 2. Materials and Methods

### 2.1. Study Design and Patient Population

This single‐center retrospective cohort study enrolled consecutive patients diagnosed with hospital‐acquired LRTI caused by MDR organisms between January 2018 and December 2023. Consecutive case inclusion was used to reduce selection bias inherent to retrospective designs. Although the single‐center design may limit external validity, it ensured consistency in diagnostic protocols and clinical management.

Multidrug resistance was defined according to the international consensus as acquired nonsusceptibility to at least one agent in three or more antimicrobial categories [12]. For key pathogens such as *A. baumannii*, *Pseudomonas aeruginosa*, and Enterobacterales, resistance patterns across clinically relevant antimicrobial categories, including carbapenems, fluoroquinolones, and aminoglycosides, were reviewed. Inclusion criteria comprised a confirmed clinical diagnosis of hospital‐acquired LRTI and a positive respiratory culture for an MDR pathogen. Exclusion criteria included polymicrobial infection and insufficient baseline laboratory data for SIRI calculation. For recurrent infections, only the first eligible episode was analyzed. After applying these criteria, 214 patients were included, with complete data for the variables used in the main analyses. The study protocol was approved by the Institutional Review Board and Ethics Committee of the hospital, and individual informed consent was waived because of the retrospective design.

### 2.2. Standardized Diagnostic Criteria

Hospital‐acquired LRTI was defined according to the IDSA/ATS guidelines, encompassing both hospital‐acquired pneumonia (HAP) and ventilator‐associated pneumonia (VAP). HAP was identified as pneumonia occurring 48 h or more after admission in nonventilated patients whereas VAP occurred after a similar duration following endotracheal intubation. Both conditions required the presence of a new pulmonary infiltrate accompanied by systemic evidence of infection such as fever, purulent sputum, and leukocytosis. For ventilator‐associated tracheobronchitis, we utilized the TAVeM study criteria requiring at least two clinical signs including abnormal body temperature, elevated white blood cell counts, or purulent secretions. The diagnosis further required microbiological confirmation from endotracheal aspirates or bronchoalveolar lavage in the specific absence of new pulmonary infiltrates on chest imaging.

### 2.3. Data Collection and Definitions

Data were extracted from electronic medical records, including demographics, comorbidities, disease severity variables, microbiological findings, and laboratory results at the time of LRTI diagnosis. The primary endpoint was all‐cause in‐hospital mortality. For survival analysis, time‐to‐event was defined from the date of MDR‐LRTI diagnosis to death or discharge. Patients discharged alive were treated as right‐censored at discharge. SIRI was calculated from the complete blood count obtained at LRTI diagnosis using the following formula: SIRI =  (peripheral neutrophil count x peripheral monocyte count)/peripheral lymphocyte count, with all cell counts expressed as x10^9^/L.

### 2.4. Statistical Analysis

Statistical analyses were conducted using R software (Version 4.2) and SPSS Statistics for Macintosh, Version 26 (IBM Corp., Armonk, NY, USA). Statistical significance was defined as a two‐sided *p* value < 0.05.

Cox proportional hazards models were used to estimate hazard ratios (HRs) and 95% confidence intervals (CIs) for the association between SIRI and in‐hospital mortality. Potential confounding variables for multivariable analysis were selected a priori according to clinical relevance, previous literature, and availability in the dataset, rather than by automated data‐driven selection. The adjusted model included age, sex, major comorbidities (diabetes mellitus, interstitial lung disease, COPD, solid malignancy, cardiovascular disease, hematologic disorder, chronic kidney disease, and chronic liver disease), and SOFA score at diagnosis. These variables were chosen because they plausibly influence both baseline inflammatory status and mortality risk. The proportional hazards assumption was assessed using Schoenfeld residuals.

Restricted cubic splines (RCSs) with four knots at the 5th, 35th, 65th, and 95th percentiles of the SIRI distribution were used to evaluate potential nonlinear associations. A data‐driven SIRI cutoff was identified from the region where the mortality HR increased on the RCS curve and was then assessed using Kaplan–Meier survival curves and log‐rank tests. The discriminative ability of SIRI as a continuous variable was further evaluated using receiver operating characteristic (ROC) curves and area under the curve (AUC) values in the overall cohort and within threshold‐defined strata. Forest plots, RCS plots, Kaplan–Meier plots, and ROC plots were generated using the CNSknowall platform based on R software.

## 3. Results

### 3.1. Baseline Clinical and Microbiological Characteristics

The study cohort comprised patients diagnosed with MDR hospital‐acquired LRTI. Of the total population, 58.4% were treated in the intensive care unit (ICU) at the time of diagnosis, while 41.6% were located in general respiratory or medical wards. Regarding the type of infection, 42.1% of cases were classified as VAP, with the remainder being nonventilated HAP. The median SOFA score at diagnosis was 4 (IQR: 2–7), reflecting a moderate‐to‐high baseline severity.

Microbiological analysis identified *A. baumannii* (36.8%), *Klebsiella pneumoniae* (29.5%), and *P. aeruginosa* (18.2%) as the predominant MDR pathogens. High rates of carbapenem resistance were observed across these species, with 84.2% of *A. baumannii* isolates being carbapenem‐resistant (CRAB). Initial antimicrobial therapy was adjudicated as appropriate—defined as the administration of at least one agent with in vitro activity against the isolated pathogen within 24 h of culture collection—in 76.3% of the cohort. Patients receiving delayed or inappropriate therapy exhibited a trend toward higher SIRI values, although this did not reach statistical significance in our descriptive comparison.

### 3.2. Association Between SIRI and Mortality

In the 214‐patient cohort, continuous SIRI was not significantly associated with mortality in the univariate Cox model (crude HR: 1.00, 95% CI: 0.99–1.00, *p* = 0.41). Similarly, after adjustment for age, sex, comorbidities, and SOFA score, continuous SIRI remained nonsignificant (adjusted HR: 1.00, 95% CI: 0.99–1.00, *p* = 0.62) (Table 1). These findings indicate that each unit increase in SIRI did not translate into a constant linear increase in mortality risk across the full observed range.

**TABLE 1:** Univariate and multivariate Cox proportional hazards regression analysis of factors associated with in‐hospital mortality.

Variable	Group	*N*	HR (95% CI)	*p*‐value	*p* for interaction
*Overall*		*214*	*2.85 (1.63–4.98)*	*<* *0.001*	
Age	≤ 71 years	113	4.11 (1.65–10.23)	0.002	0.315
	> 71 years	101	2.09 (1.01–4.32)	0.047	

Gender	Female	66	2.78 (0.98–7.86)	0.054	0.981
	Male	148	2.89 (1.45–5.76)	0.003	

SOFA score	≤ 8	134	3.52 (1.61–7.70)	0.002	0.452
	> 8	80	2.31 (0.98–5.46)	0.056	

Diabetes mellitus	No	163	3.50 (1.71–7.15)	< 0.001	0.298
	Yes	51	1.91 (0.74–4.94)	0.181	

Interstitial lung disease (ILD)	No	196	2.61 (1.45–4.70)	0.001	0.583
	Yes	18	4.99 (0.61–40.81)	0.134	

COPD	No	191	2.81 (1.58–5.01)	< 0.001	0.887
	Yes	23	3.32 (0.42–26.13)	0.255	

Solid malignancies	No	148	2.96 (1.46–5.99)	0.003	0.842
	Yes	66	2.64 (1.02–6.83)	0.045	

Cardiovascular disease (CVD)	No	98	4.51 (1.62–12.55)	0.004	0.113
	Yes	116	1.99 (1.01–3.93)	0.047	

Hematologic disorders	No	201	4.15 (2.18–7.89)	< 0.001	0.018
	Yes	13	0.11 (0.01–0.96)	0.046	

Chronic kidney disease (CKD)	No	183	3.21 (1.65–6.24)	< 0.001	0.391
	Yes	31	1.99 (0.65–6.13)	0.228	

Chronic liver disease (CLD)	No	203	3.24 (1.76–5.96)	< 0.001	0.312
	Yes	11	1.34 (0.22–8.13)	0.749	

*Note:* The multivariable model was adjusted for age, sex, diabetes mellitus, interstitial lung disease, COPD, solid malignancy, cardiovascular disease, hematologic disorder, chronic kidney disease, chronic liver disease, and SOFA score.

Abbreviations: CI, confidence interval; HR, hazard ratio; SE, standard error; SOFA, Sequential Organ Failure Assessment; SIRI, Systemic Inflammation Response Index.

This nonsignificant continuous analysis did not conflict with the subsequent threshold analysis. Rather, it suggested that the association between SIRI and mortality was not well represented by a linear model. Age remained an independent predictor of mortality in the adjusted model (adjusted HR: 1.02, 95% CI: 1.01–1.04, *p* < 0.01), whereas the SOFA score did not reach statistical significance in the fully adjusted model. Because several comorbidity strata were small, these covariate‐specific estimates should be interpreted cautiously.

### 3.3. Nonlinear Curve With a Post‐Threshold Increase and Saturation Plateau

**FIGURE 1:**
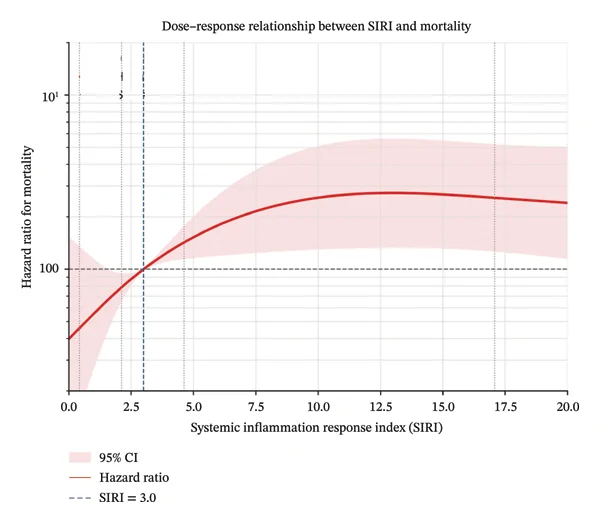
Dose–response relationship between the Systemic Inflammation Response Index (SIRI) and mortality risk. The solid red line depicts the hazard ratio (HR), while the shaded region signifies the 95% confidence interval (CI) as determined by the restricted cubic spline (RCS) model. The horizontal dashed line at HR = 1 serves as the reference for no effect. Vertical dotted lines indicate the knot locations.

RCS analysis demonstrated a significant nonlinear association between SIRI and mortality (*p* for nonlinearity < 0.001). The curve suggested a low‐risk range below approximately 3, followed by a sharp increase in mortality risk after this threshold and a subsequent plateau at higher SIRI values (Figure 1).

The lowest estimated mortality risk occurred at a SIRI value of approximately 2.97. Beyond a value of about 3.06, the lower bound of the 95% CI exceeded 1.0, suggesting a higher‐risk range. Internal validation using 1000 bootstrap resamples showed that the threshold around 3.0 was stable across resampled datasets, supporting its exploratory use for initial risk stratification in this cohort.

### 3.4. Survival Analysis Based on the Data‐Driven Cutoff

**FIGURE 2:**
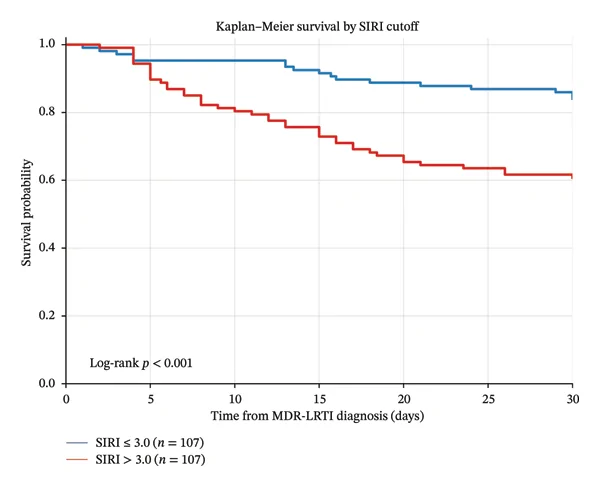
Kaplan–Meier survival curves for mortality based on the data‐driven SIRI cutoff value of 3.0. Patients were categorized into a low‐risk group (SIRI ≤ 3.0) or a high‐risk group (SIRI > 3.0). The separation of the survival curves (*p* < 0.001) supports the prognostic relevance of this threshold in the study cohort.

Based on the RCS‐derived threshold, patients were divided into a low‐risk group (SIRI ≤ 3.0, *n* = 107) and a high‐risk group (SIRI > 3.0, *n* = 107). Kaplan–Meier survival analysis showed significantly lower survival in the high‐risk group than in the low‐risk group (log‐rank *p* < 0.001) (Figure 2).

The low‐risk group had consistently higher survival probability during follow‐up, whereas the high‐risk group showed a more rapid decline. These findings support the threshold‐based interpretation of SIRI, while also indicating that this cutoff should be considered exploratory until externally validated.

### 3.5. Predictive Performance and Threshold Validation of SIRI

**FIGURE 3:**
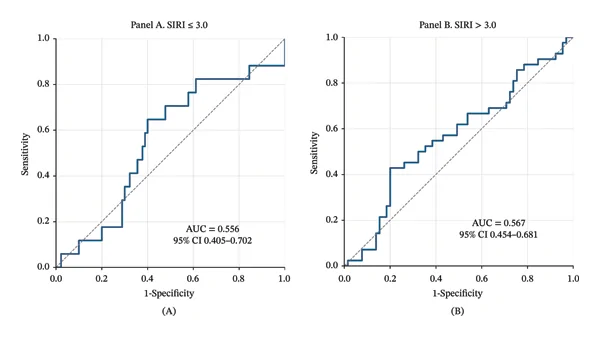
Receiver operating characteristic (ROC) curves evaluating the predictive accuracy of SIRI within threshold‐defined strata. Panel (A) shows the ROC curve for the low‐risk group (SIRI ≤ 3.0), with an AUC of 0.556. Panel (B) shows the ROC curve for the high‐risk group (SIRI > 3.0), with an AUC of 0.567. These AUC values indicate limited continuous discrimination within each stratum.

ROC analysis was used as a secondary exploratory assessment of discrimination. Although SIRI helped separate patients into risk groups at the threshold of 3.0, its continuous predictive accuracy within the threshold‐defined groups was limited. The low‐risk group (SIRI ≤ 3) had an AUC of 0.556 (95% CI: 0.407–0.706), and the high‐risk group (SIRI > 3) had an AUC of 0.567 (95% CI: 0.454–0.681) (Figure 3). These modest AUC values indicate that SIRI should be interpreted as a preliminary risk‐phenotyping marker rather than a high‐precision continuous predictor.

## 4. Discussion

In this study, a threshold‐based elevation in SIRI was associated with increased in‐hospital mortality among patients with MDR hospital‐acquired LRTI. The key finding is not that SIRI functions as a precise continuous predictor, but that a SIRI value around 3 may identify a subgroup with higher mortality risk. This interpretation is supported by the nonlinear RCS curve, the Kaplan–Meier separation between threshold‐defined groups, and the modest ROC performance within each stratum. Therefore, SIRI may be best viewed as an exploratory risk phenotype marker rather than as a stand‐alone prognostic model.

Our findings contribute to the growing literature on inflammatory indices for risk stratification in infectious diseases. Traditional scores such as APACHE II and SOFA remain important but depend on multiple physiologic and laboratory variables [4]. SIRI offers a simpler measure of host immune–inflammatory imbalance by combining neutrophils, monocytes, and lymphocytes. Previous studies have examined SIRI in malignancy and cardiovascular disease [7, 8] and in infection‐related complications after neurologic injury [9–11]. In sepsis cohorts, SIRI has also been associated with short‐ and long‐term mortality [13, 14].

However, reported SIRI thresholds vary substantially across diseases, outcomes, and statistical methods. In oncology studies, cutoffs are often derived within individual cohorts to stratify long‐term survival risk, whereas studies of sepsis or critical illness more commonly evaluate SIRI as a continuous variable, by quartiles, or through model‐based risk curves [7, 13, 14]. Studies focused on poststroke or posthemorrhage pneumonia have used SIRI mainly to predict infectious complications rather than mortality after established MDR infection [9–11]. Therefore, the cutoff of approximately 3.0 observed in the present study should not be interpreted as a universal SIRI threshold. Instead, it provides a disease‐ and cohort‐specific estimate that requires external validation before being applied beyond this dataset.

The observed nonlinear pattern is biologically plausible. At lower SIRI values, the balance between innate and adaptive immune activity may be relatively preserved. Once SIRI exceeds the threshold region, the combination of neutrophilia, monocytosis, and lymphopenia may reflect a transition toward harmful systemic inflammation and immune dysregulation. At very high values, the plateau in estimated risk may indicate biological saturation, where further increases in circulating inflammatory cells no longer produce a proportional increase in short‐term mortality risk. This interpretation is consistent with current concepts of sepsis pathophysiology, in which hyperinflammation and immunosuppression coexist [15, 16].

The potential clinical implications of these findings should be interpreted cautiously. SIRI is attractive because it is inexpensive and routinely available from complete blood counts. Nevertheless, the modest ROC performance indicates that SIRI alone is not sufficiently accurate for individual‐level clinical decision‐making. If validated in larger prospective cohorts, SIRI > 3.0 could be explored as one component of a broader risk assessment framework, rather than as an automated trigger for intervention by itself.

Several limitations should be acknowledged. First, this was a retrospective single‐center study, and residual confounding cannot be excluded. Second, some covariate strata were small, which may have reduced the stability of subgroup and multivariable estimates. Third, we did not directly compare SIRI with other inflammatory biomarkers such as procalcitonin or C‐reactive protein or with comprehensive pneumonia scores such as CPIS [17]. Finally, the proposed cutoff was derived internally and should be considered hypothesis‐generating. Future studies should externally validate the threshold, assess its performance in multicenter cohorts, and evaluate whether dynamic changes in SIRI add prognostic information.

## 5. Conclusion

SIRI may provide an accessible marker for preliminary mortality risk stratification in patients with MDR hospital‐acquired LRTI. In this cohort, the prognostic information of SIRI appeared to be threshold‐based rather than linearly continuous, with a data‐driven cutoff around 3.0 identifying patients at higher risk. However, because discrimination was modest and the study was retrospective and single‐center, SIRI should not be used as a stand‐alone clinical decision tool. Prospective multicenter validation is needed before this cutoff can be recommended for routine clinical application [18–23].

## Author Contributions

The study was conducted and written by Haixing Zhu and Jiebai Zhou. Xia Hui and Jiebai Zhou conducted the statistical analyses. Xia Hui, Yahui Liu, and Xiao Ge contributed patient information. Jiebai Zhou revised the manuscript. Haixing Zhu conceptualized the research.

## Funding

This research received funding from Science and Technology Program of Gansu Province (Grant 26JRRA320), the National Natural Science Foundation of China (Grants 81600014 and 82170086), the Shanghai Key Laboratory of Emergency Prevention (Grant 20dz226110), the Shanghai Municipal Key Clinical Specialty (Grant shslczdzk02202), and the Cultivation Project of Shanghai Major Infectious Disease Research Base (Grant 20dz221050).

## Disclosure

All authors reviewed and approved the final version of the manuscript.

## Ethics Statement

The protocol for this study was reviewed and approved by the Ruijin Hospital Ethics Committee at Shanghai Jiao Tong University School of Medicine. Due to the retrospective nature of the study, the requirement for obtaining written informed consent from all participants was waived by the Ethics Committee. All patient data were anonymized prior to analysis to ensure confidentiality and privacy.

## Conflicts of Interest

The authors declare no conflicts of interest.

## Supporting Information

Additional supporting information can be found online in the Supporting Information section.

## Supporting information


**Supporting Information** Supporting Data File 1: Raw data for the restricted cubic spline (RCS) analysis. This data file contains the underlying values used to generate the dose–response relationship curve between the Systemic Inflammation Response Index and mortality risk, as presented in Figure 1 of the main manuscript. The file includes calculated hazard ratios, 95% confidence intervals, and knot locations used for the RCS model.

## Data Availability

The deidentified dataset utilized in this study is not publicly accessible in accordance with institutional regulations safeguarding patient confidentiality. Nonetheless, anonymized data can be provided to eligible researchers upon request, pending approval by the Institutional Review Board (IRB) of Ruijin Hospital Affiliated with Shanghai Jiao Tong University School of Medicine. Requests for data access should be submitted to hospitaloffice@rjh.com.cn, accompanied by a comprehensive research proposal and adherence to data protection protocols.
